# B-Mode and Doppler Ultrasonographic Changes in the Corpus Luteum, Uterus, and Uterine Artery During Early Pregnancy in Cows

**DOI:** 10.3390/life16030378

**Published:** 2026-02-27

**Authors:** Murat Can Demir, Merve Sena Demir, Burak Büyükbaki, Mushap Kuru, Semra Kaya, Cihan Kaçar

**Affiliations:** 1 Department of Obstetrics and Gynecology, Faculty of Veterinary Medicine, Kafkas University, 36100 Kars, Türkiye; 2 Department of Wildlife Diseases and Ecology, Faculty of Veterinary Medicine, Kafkas University, 36100 Kars, Türkiye

**Keywords:** bovine, corpus luteum, Doppler ultrasonography, early pregnancy, echotexture analysis, luteal blood perfusion

## Abstract

The aim of this study was to comparatively evaluate echotextural and hemodynamic changes in the corpus luteum (CL), uterus, and uterine artery, together with serum progesterone (P4) concentrations, using B-mode and Doppler ultrasonography between days 5 and 21 post-insemination in pregnant and non-pregnant cows. Twelve clinically healthy Brown Swiss cows were enrolled and allocated into a cyclic non-inseminated group (*n* = 6) and an inseminated group (*n* = 6). Ultrasonographic examinations and progesterone measurements were performed daily during the post-insemination period, and pregnancy was confirmed on day 30. Echotextural parameters (mean gray value and homogeneity) were obtained from the corpus luteum and uterus using B-mode ultrasonography. Doppler ultrasonography was used to assess corpus luteum vascular parameters and uterine artery blood flow, and serum progesterone concentrations were measured at each examination. Corpus luteum mean gray value showed a significant time effect (*p* < 0.001). For Corpus luteum area and perfusion area, both the time effect and the group × time interaction were significant (*p* < 0.001), and marked differences between pregnant and non-pregnant cows were observed on days 19, 20, and 21 (*p* < 0.05). Serum progesterone concentrations also differed significantly between groups on days 20 and 21. In conclusion, changes in corpus luteum area and perfusion area were associated with early pregnancy-related differences and may represent earlier functional ultrasonographic indicators compared with uterine artery Doppler parameters and progesterone concentrations alone. These findings may have practical implications for herd management by potentially enabling differentiation between pregnant and non-pregnant cows approximately 1–2 days earlier than serum progesterone measurements.

## 1. Introduction

Pregnancy diagnosis is a critical step in cattle reproductive management. Early identification of non-pregnant females is important for the timely implementation of production-related decisions, such as re-insemination or culling of these animals [1]. The earlier that pregnancy can be diagnosed, the more feasible it becomes to optimize reproductive performance and increase economic efficiency. Therefore, the development of an early, accurate, and practical pregnancy diagnosis method is of great importance [2].

During the pre-implantation period, the uterus undergoes a series of physical and biochemical changes in preparation for implantation. Secretions released from the uterine glandular epithelium provide the nutrients and hormones required for the conceptus to survive until implantation [3]. These morphological changes become ultrasonographically visible in the later stages of pregnancy with the presence of the fetus [4]. Pregnancy diagnosis based on ultrasonographic visualization of the embryonic vesicle is generally not recommended before days 26 to 28 of gestation due to false-positive evaluations [4,5]. To overcome these limitations, the inclusion of technologies such as Doppler ultrasonography, in addition to embryonic vesicle visualization using B-mode ultrasonography, allows a more detailed evaluation of the uterus, ovarian follicles, and corpus luteum. Color Doppler mode enables visualization of blood flow within tissues and structures, thereby allowing functional assessment of reproductive tissues beyond morphological evaluation. In cattle reproduction, this approach has been increasingly applied to evaluate luteal vascularization and early functional changes associated with pregnancy establishment [6,7]. Although uterine artery Doppler indices have been proposed as early indicators of pregnancy, evidence remains inconsistent, particularly during the pre-implantation period.

Recent advances in reproductive ultrasonography, particularly the integration of color Doppler imaging and computer-assisted echotexture analysis, have enabled detailed functional assessment beyond conventional morphological evaluation. In cattle, color and spectral Doppler ultrasonography have increasingly been used to assess luteal blood flow as an indirect indicator of corpus luteum functionality and progesterone production [8,9]. Several studies have suggested that increased luteal vascularization may serve as an early marker of pregnancy maintenance before embryonic structures become detectable using conventional B-mode imaging [10,11]. In addition to vascular assessment, computer-assisted echotexture analysis has emerged as a complementary approach for evaluating structural changes in reproductive tissues, including alterations in gray-scale intensity and tissue homogeneity associated with luteal development and regression [12,13]. Using these methods, endometrial and luteal changes can be identified in cows during the early pregnancy period and throughout the cycle [12]. In addition, in veterinary medicine, these techniques make significant contributions to understanding the migration of the conceptus within the uterus, ovulation, endometrial vascularization during embryonic and placental development, determination of fetal sex, and changes in endometrial echotexture during the cycle and implantation [14,15].

However, most previous studies have focused on individual ultrasonographic parameters or limited time points rather than continuous longitudinal evaluation during the early post-insemination period [16,17]. To our knowledge, no previous study has performed a simultaneous daily longitudinal evaluation of luteal echotexture, luteal perfusion, uterine echotexture, and uterine arterial hemodynamics within a single experimental framework during the critical window of maternal recognition of pregnancy. Although uterine artery Doppler indices have been proposed as potential early indicators of pregnancy, findings remain inconsistent, particularly during the pre-implantation period [18]. This inconsistency highlights an ongoing debate regarding whether early pregnancy-related adaptations are predominantly detectable through luteal functional changes or whether measurable macrovascular alterations occur simultaneously within the uterine circulation. Clarifying whether early pregnancy-related adaptations are primarily localized at the luteal level or reflected in broader uterine hemodynamic changes is essential for improving the interpretation of ultrasonographic markers during early gestation. Based on this rationale, we hypothesized that early pregnancy-related adaptations would be predominantly detectable through luteal structural and vascular parameters rather than uterine macrovascular Doppler indices. Accordingly, the aim of this study was to perform a daily longitudinal comparative evaluation of echotextural and hemodynamic changes in the corpus luteum, uterus, and uterine artery between days 5 and 21 after insemination in pregnant and non-pregnant cows using B-mode and Doppler ultrasonography, together with serum progesterone (P4) measurements, to investigate early pregnancy-related luteal maintenance and associated uterine hemodynamic adaptations.

## 2. Materials and Methods

### 2.1. Ethical Approval

The study was conducted in accordance with the approval obtained from the Kafkas University Local Ethics Committee for Animal Experiments (KAU-HADYEK/2023-147) and within the framework of the relevant ethical principles.

### 2.2. Animals

This study was conducted on a total of 12 clinically healthy Brown Swiss cows with regular estrous cycles (21 days) that had calved normally in the previous lactation period and were housed at the Kafkas University Faculty of Veterinary Medicine Prof. Dr. Ali Rıza Aksoy Education, Research and Application Farm. The mean age of the animals was 4.2 ± 0.2 years (2.9–5.6), and the mean parity was 2.4 ± 0.2 (1–4), with all cows being at least 8 weeks into lactation. Body condition score (BCS) was evaluated on a scale ranging from 1 (emaciated) to 5 (obese). Only cows with a mean BCS of 3.00 ± 0.3 (2.50–3.50) were included in the study. Throughout the study period, all animals were maintained under standard management conditions and fed a uniform mixed ration consisting of grass hay, corn silage, and cattle concentrate; ad libitum access to water, salt, and a mineral mixture was provided. To ensure animal welfare and minimize stress, all handling and examinations were performed in a calm and quiet environment by an experienced operator. Animals were allowed sufficient time to acclimate after handling and before ultrasonographic procedures were initiated. Daily monitoring of general health status and behavior was performed throughout the study period.

### 2.3. Experimental Design

For ovulation synchronization, a progesterone-supported Ovsynch protocol was applied to all cows. Within the scope of the protocol, gonadotropin-releasing hormone (GnRH; 2 mL, i.m., 50 µg/mL gonadorelin diacetate tetrahydrate, Ovarelin^®^, CEVA^®^, Istanbul, Türkiye) was injected on day 0, and simultaneously, an intravaginal progesterone device (1.55 g progesterone, PRID Delta^®^, CEVA^®^) was inserted and maintained for 7 days. On day 7, concomitant with PRID removal, prostaglandin F_2_α (PGF_2_α; 5 mL, i.m., 5 mg/mL dinoprost tromethamine, Enzaprost^®^, CEVA^®^, Istanbul, Türkiye) was administered. This was followed by a second GnRH injection 48 h later. For estrus detection, animals were observed four times daily. Cows exhibiting estrus were randomly assigned to two groups: an inseminated group (*n* = 6) and a non-inseminated cyclic group (*n* = 6). Group allocation was performed using a computer-generated randomization procedure to minimize allocation bias. Ovulation was confirmed by B-mode ultrasonographic examinations (5–7.5 MHz linear probe, Draminski iScan, Sząbruk, Poland) performed at 12 h intervals, and the day on which the dominant follicle was first no longer detectable was accepted as day 1 of the estrous cycle.

To evaluate echotextural and hemodynamic changes following artificial insemination, B-mode and color Doppler ultrasonographic examinations of the ovaries, uterus, and uterine artery were performed daily between days 5 and 21. During ultrasonographic imaging, in both pregnant and non-pregnant animals, only data obtained from the side where the corpus luteum was localized were recorded and evaluated in order to ensure physiological comparability. Ultrasonographic assessments were performed at similar times of day for each animal, and the total duration of procedures, including imaging and blood sampling, was limited to 30–45 min. In the inseminated group, pregnancy status was confirmed on day 30 by ultrasonographic detection of embryonic cardiac activity.

### 2.4. Ultrasonographic Examination

For B-mode and Doppler ultrasonographic imaging, an ESAOTE MyLab™ DeltaVET (Esaote Biomedica^®^, Genova, Italy) ultrasound system equipped with a 5–10 MHz multifrequency linear transrectal probe was used. To ensure image standardization and allow reliable comparisons between examinations, device settings were kept constant throughout all imaging procedures and across all anatomical sites evaluated (imaging depth: 6 cm, focal zone: 2.5 cm, gain: 70%, PRF: 1 kHz, frequency: 8–10 MHz). During pulsed-wave Doppler measurements, the insonation angle was maintained between 20° and 50°, and the sample volume was set to 1 mm. All ultrasonographic examinations were performed by the same operator.

### 2.5. Echotexture Analysis

**Figure 1:**
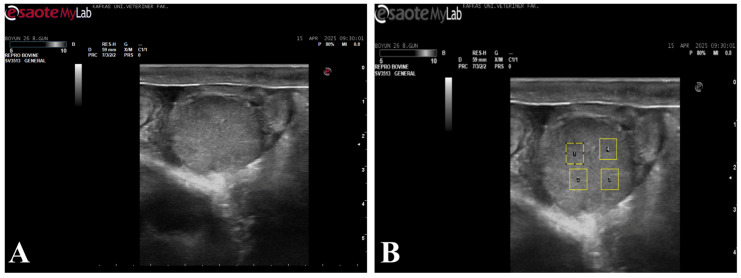
B-mode ultrasonographic image of the corpus luteum used for echotexture analysis. (**A**) B-mode ultrasonographic image of the corpus luteum; (**B**) The same image showing the placement of four regions of interest (ROIs) selected for ImageJ-based grayscale and homogeneity analysis.

For echotexture analysis, the recorded images were transferred to a digital environment in bitmap (BMP) format and converted to grayscale prior to analysis. The images were analyzed using ImageJ (v. 1.52, NIH, ABD) software. In each image, four artifact-free regions of interest (ROIs; 35 × 35 pixels) (Figure 1) covering the ovary and endometrium were defined. ROI size was selected in accordance with previously published echotextural analyses employing similar ROI dimensions for standardized image evaluation [12,19]. For corpus luteum analysis, ROIs were placed within homogeneous luteal parenchyma while avoiding cavitations, peripheral borders, and acoustic shadowing artifacts. For uterine assessment, ROIs were positioned within the endometrial layer at comparable anatomical locations, avoiding luminal fluid, folds, and artifacts. ROI placement was performed using consistent anatomical landmarks and standardized image planes to ensure repeatability between examinations. A total of 12 ROIs were evaluated for each animal. Corpus luteum mean gray value (CL-MGV), uterine mean gray value (UT-MGV), as well as corpus luteum homogeneity (CL-HOM) and uterine homogeneity (UT-HOM) parameters, were calculated and used in the statistical analyses. Image analysis and parameter extraction were performed by the same investigator using standardized procedures. Blinding to group allocation was not applied due to the nature of repeated ultrasonographic measurements; however, consistent acquisition settings and predefined analysis protocols were used to minimize potential observer bias.

### 2.6. Perfusion and Spectral Doppler Analysis

**Figure 2:**
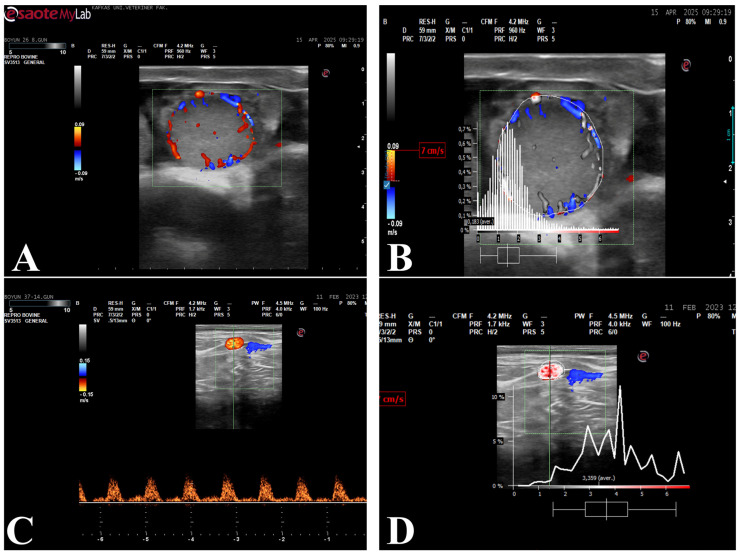
Color Doppler ultrasonographic evaluation of the corpus luteum and spectral Doppler assessment of uterine artery blood flow. (**A**) Color Doppler illustrating luteal perfusion area; (**B**) Analysis of the perfusion area within the identified corpus luteum; (**C**) Spectral Doppler of the uterine artery; (**D**) Spectral Doppler waveform analysis obtained from the uterine artery.

Perfusion of the corpus luteum and spectral Doppler analyses of the uterine artery were performed using PixelFlux (v. 18.03.11, Chameleon® Software, Münster, Germany) software. Corpus luteum perfusion area (CL-PA) and corpus luteum area (CL-A) were calculated based on the mean values obtained from three separate images. The three images were acquired consecutively during the same examination session, and frames representing the maximal cross-sectional area of the corpus luteum with clearly visible vascular signals were selected to ensure measurement consistency. Care was taken to avoid artifacts and frames with incomplete vascular visualization. From the spectral Doppler waveforms obtained from the uterine artery, maximum velocity, blood flow average velocity, blood flow volume, resistance index, uterine artery area and pulse rate were calculated. Measurements were performed on images containing three regular cardiac cycles (Figure 2).

### 2.7. Hormone Analysis

Following ultrasonographic examinations, blood samples collected from the coccygeal vein were centrifuged at 3500 rpm for 10 min to separate the serum, which was then stored at −20 °C until analysis. Quantitative serum progesterone (P4) measurements were conducted using the direct chemiluminescence immunoassay (CLIA) method. The analyses were performed using a Siemens hormone analyzer and ADVIA Centaur^®^ test kits (Siemens, Tarrytown, NY, USA). The ADVIA Centaur test demonstrated a sensitivity of 100% and specificity of 95.5%. According to the manufacturer’s validation data, intra-assay and inter-assay coefficients of variation were <4.39% and <2.60%, respectively.

### 2.8. Statistical Analysis

A post hoc power analysis was performed using G*Power software (Version 3.1.9.7, Düsseldorf, Germany). Assuming a repeated-measures design with 17 measurements, a medium effect size (f = 0.25), an α error probability of 0.05, and a correlation among repeated measures of 0.5, the achieved statistical power with 14 subjects was estimated to be approximately 0.85.

Statistical analyses of the data were performed using SPSS version 26.0 (IBM Corp., Armonk, NY, USA). Descriptive statistics for the parameters according to days and groups were expressed as mean ± standard error of the mean (SEM). Normality was assessed using the Shapiro–Wilk test. For all parameters, the effects of pregnancy (group effect), time (time effect), and group × time interaction were evaluated. For this purpose, repeated-measures analysis of variance was used. When the assumption of sphericity was not met, the Green-house–Geisser correction was applied. If the group × time interaction was significant, pair-wise comparisons with Bonferroni correction were used to determine at which time points differences occurred between groups. Additionally, the effect size for each factor and their interaction was determined by calculating the partial eta squared (η_p_^2^). The magnitude of the effect was interpreted as small (0.01 to 0.06), medium (0.06 to 0.14), or large (>0.14) based on established benchmarks. Pearson correlation coefficients (r) were reported to assess relationships between selected variables. These correlation analyses were considered exploratory to identify potential associations between ultrasonographic and hormonal parameters. In all analyses, *p* < 0.05 was considered statistically significant.

## 3. Results

### 3.1. Ultrasonographic Changes in the Corpus Luteum and Uterus

**Figure 3:**
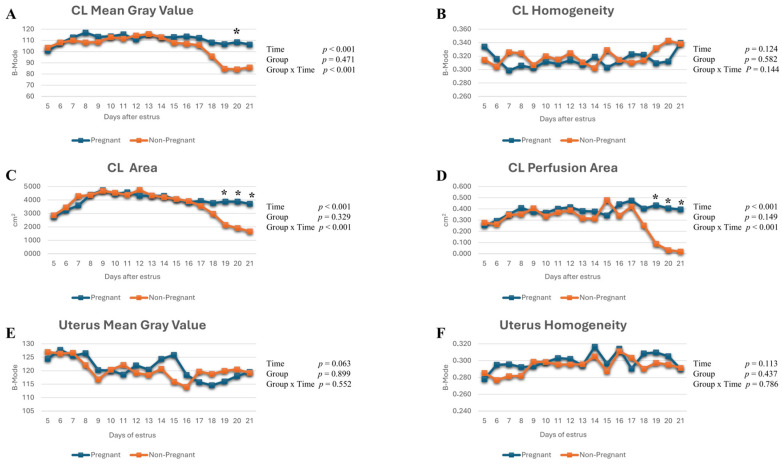
(**A**–**D**) Changes in corpus luteum mean gray value, homogeneity, area, and perfusion area post-insemination; (**E**,**F**) changes in uterine mean gray value and homogeneity post-insemination. *: indicates statistically significant differences between days in pregnant and non-pregnant cows, *p* < 0.005.

CL-MGV showed a significant time effect (*p* < 0.001, η_p_^2^ = 0.486) and group × time interaction (*p* < 0.001, η_p_^2^ = 0.281). Pregnant cows exhibited higher CL-MGV values than non-pregnant cows on day 20 post-insemination (*p* = 0.030) (Figure 3A).

No significant time-dependent changes were detected for CL-HOM (η_p_^2^ = 0.150), UT-MGV (η_p_^2^ = 0.185), or UT-HOM (η_p_^2^ = 0.152), and no significant group × time interaction was observed for these parameters (η_p_^2^ = 0.144, 0.074, and 0.050, respectively; *p* > 0.05). In addition, no statistically significant differences were identified between pregnant and non-pregnant cows for these parameters throughout the study period (*p* > 0.05) (Figure 3B,E,F).

CL-A and CL-PA demonstrated significant time effects (*p* < 0.001, η_p_^2^ = 0.696 and 0.312, respectively) and group × time interactions (η_p_^2^ = 0.462 and 0.330, respectively). These parameters diverged between groups on days 19, 20, and 21 post-insemination (*p* < 0.05, *p* < 0.001, and *p* < 0.001, respectively) (Figure 3C,D).

### 3.2. Ultrasonographic Changes in the Uterine Artery

**Figure 4:**
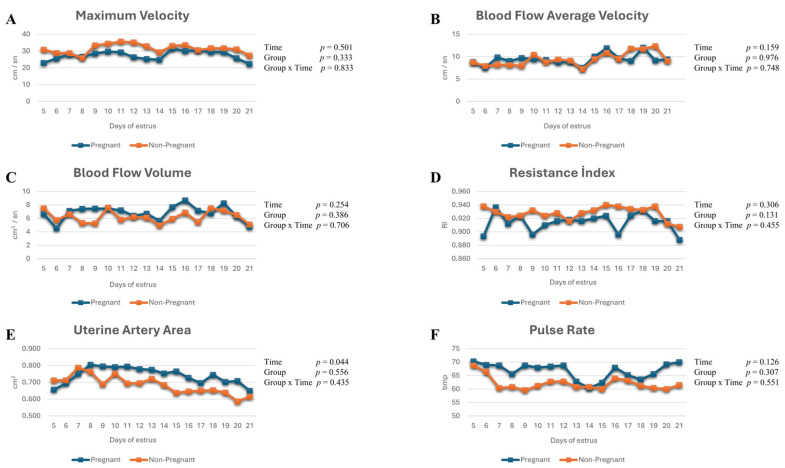
(**A**–**F**) Uterine artery maximum velocity, blood flow average velocity, blood flow volume, resistance index, uterine artery area, and pulse rate values in pregnant and non-pregnant cows.

No significant time effects were detected for uterine artery maximum velocity (η_p_^2^ = 0.074), mean blood flow velocity (η_p_^2^ = 0.144), resistance index (η_p_^2^ = 0.110), or pulse rate (η_p_^2^ = 0.215) throughout the study period (*p* > 0.05). Likewise, no significant group × time interactions (η_p_^2^ = 0.027, 0.050, 0.087, and 0.088, respectively) or between-group differences were observed for these parameters, indicating that uterine artery spectral Doppler indices remained largely stable during early pregnancy (Figure 4A–D,F).

Although a statistically significant time-dependent change was observed in uterine artery area (*p* = 0.044, η_p_^2^ = 0.120), the group × time interaction was not significant (*p* = 0.435, η_p_^2^ = 0.056), suggesting that this variation was not pregnancy-specific (Figure 4E).

### 3.3. Changes in Serum Progesterone Concentrations

**Figure 5:**
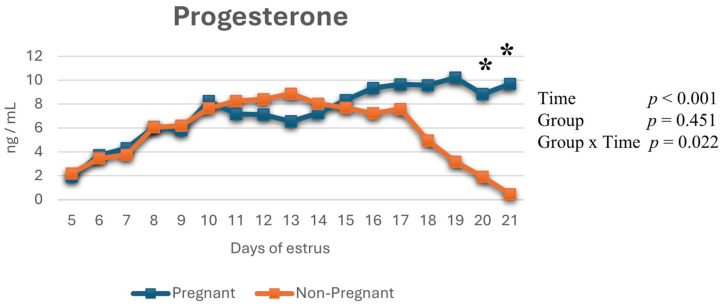
Changes in serum progesterone (P4) concentrations throughout the study period. *: indicates statistically significant differences between days in pregnant and non-pregnant cows (*p* < 0.005).

P4 concentrations showed a statistically significant time-dependent change (*p* < 0.001, η_p_^2^ = 0.398), and a significant group × time interaction was observed (*p* = 0.022, η_p_^2^ = 0.268). Specifically, on days 20 and 21 after estrus, serum P4 differed significantly between pregnant and non-pregnant cows (*p* < 0.05), with higher concentrations observed in the pregnant group. However, the overall group effect did not reach statistical significance (*p* = 0.451, η_p_^2^ = 0.058) (Figure 5).

### 3.4. Correlations Between Parameters

Pearson correlation coefficients among corpus luteum-related variables, as well as uterine, uterine artery, and hemodynamic parameters, are presented in Table 1. In pregnant cows, significant positive correlations were observed among CL-MGV, CL-A, and CL-PA, as well as between CL-related parameters and UT-MGV (*p* < 0.01). Progesterone concentrations were positively correlated with CL-MGV (r = 0.237, *p* = 0.016) and CL-A (r = 0.288, *p* = 0.003). No significant correlations were detected between progesterone and uterine parameters (UT-MGV, uterine homogeneity) or uterine artery Doppler parameters, including uterine artery area, maximum velocity, mean velocity, flow volume, resistance index, and pulse number (*p* > 0.05). No significant correlation was detected between CL-PA and P4.

In non-pregnant cows, similar positive relationships were identified among CL structural and vascular parameters, with additional correlations between CL-A and P4 and a negative correlation between CL-PA and P4 (*p* < 0.05–0.01). Progesterone was negatively correlated with UT-MGV (r = −0.366, *p* < 0.001) and CL homogeneity (r = −0.271, *p* = 0.006) and positively correlated with maximum velocity (r = 0.362, *p* < 0.001) and resistance index (r = 0.279, *p* = 0.004). A negative correlation was observed between progesterone and pulse number (r = −0.262, *p* = 0.008).

## 4. Discussion

The main novelty of this study lies in the daily evaluation of ultrasonographic parameters of the corpus luteum, uterus, and uterine artery in pregnant and non-pregnant cows during the post-insemination period, and in the comprehensive demonstration of the relationships between these parameters and progesterone in early pregnancy. In the early stages of pregnancy, molecular and immunological processes mediated by various hormones and cytokines enable maternal recognition of the embryo and the maintenance of luteal function [20]. The conceptual approach suggesting that the structural and functional adaptations accompanying these processes can be detected by ultrasonographic methods at very early stages of pregnancy constituted the basis for the interpretation of the findings obtained in the present study.

### 4.1. Evaluation of Corpus Luteum Findings

In this study, when ultrasonographic parameters of the CL were evaluated, it was observed that significant changes specific to early pregnancy emerged, particularly in CL-MGV, CL-A, and CL-PA. The presence of a significant time-dependent change in CL-MGV and the detection of a group × time interaction may reflect that luteal tissue maintains its structural integrity following maternal recognition of pregnancy. The higher CL-MGV observed in pregnant cows on day 20 post-insemination is consistent with the presence of a functional corpus luteum in which luteolysis is suppressed and progesterone synthesis is maintained [21]. This finding is consistent with previous studies reporting that luteal cell density and stromal organization are preserved in early pregnancy [8,22]. This difference on day 20 coincides with the peak of maternal recognition signaling, which may explain the transient differentiation in CL-MGV.

The significant differences in CL-A and CL-PA between pregnant and non-pregnant cows on days 19–21 post-insemination indicate that the luteal structure is preserved in a pregnancy-specific manner not only at the morphological level but also at the functional and vascular levels. The higher luteal perfusion area observed in pregnant animals may be associated with the continued vascularization of luteal tissue in response to the increased progesterone demand required to sustain embryonic development [11]. Previous studies have shown that luteal blood flow assessed by Doppler ultrasonography can serve as a sensitive indicator for early pregnancy diagnosis [10,23]. Consistent with these reports, our results indicate that evaluating corpus luteum area together with perfusion area enables a more comprehensive assessment of luteal function during early pregnancy. Similarly, Doppler-based longitudinal investigations have demonstrated that luteal blood flow remains elevated in pregnant animals while decreasing prior to luteolysis in non-pregnant cows, indicating that vascularization reflects functional luteal activity rather than merely structural persistence [18,24]. However, it should be emphasized that increased luteal perfusion and structural maintenance are not exclusively specific to pregnancy, as similar patterns may also be observed in conditions associated with prolonged luteal function or delayed luteolysis. Therefore, interpretation of luteal ultrasonographic parameters should be performed in conjunction with temporal dynamics, progesterone profiles, and overall reproductive status rather than being considered definitive standalone indicators of pregnancy. From a practical perspective, combined evaluation of CL structural and perfusion parameters may support earlier identification of non-pregnant cows and facilitate timely reproductive management decisions.

Studies performed in different cattle breeds have demonstrated that pregnancy-related differences in CL-A and CL-PA may emerge at earlier stages [16,17]. In the present study, the emergence of pregnancy-specific differences on days 19–21 may be explained by breed-related differences, as well as by variations in physiological status, individual variability, and differences in the timing associated with the evaluation methods used. In addition, variability in interferon-tau-mediated luteoprotective effects among animals may lead to individual differences in the timing of luteolysis suppression, resulting in the appearance of morphological and vascular changes in the corpus luteum at later stages.

The absence of significant differences in corpus luteum homogeneity across time or pregnancy status indicates that this parameter is more likely related to luteal maturation and cellular remodeling rather than to early pregnancy itself. Previous studies have shown that CL-HOM offers limited discriminatory value for pregnancy diagnosis and is therefore mainly regarded as a supportive parameter [7,19]. In addition, CL homogeneity varies across the estrous cycle as a consequence of dynamic changes in cellular composition. The results of the present study align with this pattern [13,25,26].

### 4.2. Evaluation of Ultrasonographic Findings of the Uterus

In this study, the absence of significant differences in UT-MGV and UT-HOM parameters with respect to time or pregnancy status during the period post-insemination may suggest that uterine changes occurring in the early stages of pregnancy take place primarily at the molecular and cellular levels. During the early phase of maternal recognition of pregnancy, the uterine response is characterized primarily by histological and immunological adaptations mediated by interferon-tau, progesterone, and various cytokines. These early responses are not necessarily accompanied by marked changes in tissue echogenicity as assessed by B-mode ultrasonography [15,21]. Accordingly, previous quantitative echotexture analyses evaluating endometrial optical density have also shown that ultrasonographic variations in uterine tissue during early pregnancy may be subtle and inconsistent, limiting their diagnostic value when used alone [12]. Functional changes within the uterine lumen, endometrial glands, and stromal cells during early pregnancy tend to become morphologically apparent only at more advanced stages of gestation [3,27]. In this context, the limited discriminatory power of uterine MGV and homogeneity for early pregnancy diagnosis supports the notion that these parameters are of secondary importance compared with ultrasonographic markers related to the luteal structure. From a practical standpoint, these findings indicate that uterine echotextural parameters alone may have limited utility for early differentiation between pregnant and non-pregnant cows.

In cyclic cows, physiological edema developing in the endometrium during different phases of the estrous cycle may lead to transient changes in intra- and extracellular fluid distribution. Because intracellular fluids are hypoechoic, periods of increased endometrial edema are generally associated with reduced echogenicity. By contrast, during diestrus, a decrease in fluid content together with increased cellular density may lead to relatively higher echogenicity [28]. Consistent with previous longitudinal ultrasonographic evaluations, physiological echotextural fluctuations do not necessarily translate into reliable ultrasonographic markers capable of distinguishing reproductive status during early pregnancy [19]. In the present study, however, the lack of significant variation in UT-MGV and UT-HOM parameters in cyclic animals indicates that these physiological fluctuations do not result in persistent structural changes detectable by ultrasonography. One of the main reasons for this may be that changes in endometrial edema and fluid distribution are largely microscopic and transient in nature. Although such fluctuations may be evident at the histological level, they may not consistently or directly influence tissue echogenicity and homogeneity as assessed by B-mode ultrasonography.

### 4.3. Evaluation of Ultrasonographic Findings of the Uterine Artery

In this study, the absence of significant differences in uterine artery spectral Doppler parameters (maximum velocity, mean blood flow velocity, blood flow volume, resistance index, and pulse rate) with respect to pregnancy status or group × time interaction may indicate that uterine hemodynamic adaptations have not yet become evident at the macrovascular level during the very early stages of pregnancy. During early pregnancy, progesterone- and interferon-tau-mediated effects are thought to operate mainly through local regulatory mechanisms at the endometrial and luteal levels, while pronounced vascular remodeling reflected in uterine artery blood flow tends to occur at more advanced stages of gestation [29]. Accordingly, marked changes in uterine artery Doppler indices are more likely to occur during later stages of pregnancy or once placentation becomes established [9,30].

In the present study, only uterine artery area exhibited a time-dependent change, indicating that vascular adaptation may begin during the early period. Nevertheless, this alteration does not yet appear to be sufficient to be captured by spectral Doppler parameters. These findings emphasize that the absence of discriminatory uterine artery Doppler changes represent an important physiological observation rather than a methodological limitation, suggesting that uterine artery Doppler measurements may provide more meaningful information during later stages, when uterine and uteroplacental circulation undergo substantial remodeling, rather than serving as primary markers for early pregnancy diagnosis.

Although these findings may appear, at first glance, to differ from early uterine blood flow changes reported in the literature [18,24], they are not physiologically or methodologically contradictory. Uterine blood flow changes observed in early pregnancy are predominantly local and transient [24]. This suggests that spectral Doppler measurements obtained from the proximal segment of the uterine artery may not always reflect early adaptations occurring at the endometrial or microvascular levels [31]. In the present study, the lack of discriminatory uterine artery spectral Doppler parameters indicates that uterine hemodynamic adaptations in early pregnancy have not yet become apparent at the macrovascular level and is consistent with the high degree of individual variability reported in the literature. From a clinical perspective, these results suggest that uterine artery Doppler indices alone may have limited utility for very early pregnancy assessment.

### 4.4. Evaluation of Progesterone Findings

In this study, the observation of a significant time-dependent change in serum P4 concentrations together with a significant group × time interaction may indicate that luteal function is dynamically regulated during early pregnancy. In particular, higher P4 levels observed in pregnant cows on days 20–21 post-insemination may suggest that luteolysis is suppressed following maternal recognition of pregnancy and that a functional corpus luteum is maintained [3,27]. Similar temporal increases in progesterone concentrations during early pregnancy have been reported in previous studies evaluating luteal function using endocrine and ultrasonographic approaches, supporting the association between sustained progesterone secretion and luteal maintenance [7,27,32].

Despite the marked increase in progesterone concentrations, the absence of significant changes in uterine artery spectral Doppler parameters suggests that early progesterone-mediated effects may remain localized rather than inducing detectable macrovascular adaptations. Comparable findings have been reported in studies assessing uterine hemodynamics during early pregnancy, where increases in progesterone were not consistently accompanied by measurable changes in uterine artery Doppler indices during the early post-insemination period [9]. Progesterone influences uterine microcirculation through mechanisms involving nitric oxide synthesis, prostaglandin balance, and local angiogenesis; however, these effects are reflected in uterine artery Doppler indices measured at the macrovascular level during more advanced stages of pregnancy [9,33]. Therefore, the strong concordance observed in this study between the increase in P4 and CL-related ultrasonographic parameters suggests that progesterone primarily acts to maintain luteal function and to prepare the uterine microenvironment during early pregnancy.

In a previous study, CL-PA was shown to be a more sensitive marker than progesterone concentration for early pregnancy assessment in cows [7,34]. Consistent with this, in the present study, differences in CL-PA and CL-A emerged one day earlier than differences in progesterone concentrations, supporting the view that luteal blood flow represents an early and dynamic indicator of luteal function [10]. This finding indicates that luteal vascular regression may reflect a decline in progesterone synthesis before structural regression becomes apparent. Taken together, these findings indicate that although progesterone remains a central biological marker of early pregnancy physiology, its interpretation is strengthened when integrated with structural and vascular ultrasonographic parameters, thereby improving early pregnancy assessment and evaluation of luteal function. From a practical perspective, this combined approach may facilitate earlier identification of non-pregnant animals and support more timely reproductive management decisions.

### 4.5. Evaluation of Correlation Findings

In this study, correlation analyses among corpus luteum (CL), uterus, uterine artery, and progesterone parameters allowed a comprehensive evaluation of the physiological interaction between luteal function and the uterine environment during early pregnancy. In pregnant cows, the positive relationship between CL area and progesterone concentration supports the concept that the morphological continuity of the luteal structure is directly linked to its steroidogenic capacity. The literature has reported a strong association between CL size and vascularization and progesterone production, with this relationship becoming more pronounced during early pregnancy [10,23].

The positive correlations between CL-MGV and CL-A and CL-PA indicate that increased echogenicity of the luteal tissue is not merely a structural feature but also represents an ultrasonographic reflection associated with functional vascular integrity. These findings are consistent with previous studies reporting that the combined evaluation of B-mode and Doppler ultrasonographic parameters contributes to a more accurate and comprehensive interpretation of luteal function [8,9].

Overall, the correlation findings demonstrate that, during early pregnancy, there is a strong and consistent relationship among progesterone concentration, structural characteristics of the CL, and luteal vascularization, whereas uterine and uterine artery parameters play a more indirect and secondary role. These results indicate that, in the evaluation of early pregnancy physiology, the combined use of CL-focused ultrasonographic parameters together with progesterone measurements provides a biologically and diagnostically more meaningful approach.

## 5. Limitations

Despite the strengths of the present study, several limitations should be acknowledged. First, although repeated longitudinal measurements were performed to enhance statistical robustness, the relatively small sample size may limit the generalizability of the findings to broader cattle populations. Second, echotexture analyses were conducted using standardized ROI selection criteria to minimize variability; however, intra- and inter-observer reliability metrics, such as intraclass correlation coefficient (ICC) or coefficient of variation (CV), were not calculated. Therefore, although efforts were made to ensure consistency in image acquisition and analysis, potential observer-related variability cannot be fully excluded. In addition, uterine artery Doppler measurements were obtained from macrovascular segments, which may not fully reflect early microvascular adaptations occurring during the maternal recognition period. Finally, the observational design of the study limits causal interpretation of correlations between ultrasonographic parameters and progesterone concentrations. Future studies including larger sample sizes, reliability assessments, and multi-operator validation are warranted to confirm and extend these findings.

## 6. Conclusions

In conclusion, this study demonstrated that uterine artery spectral Doppler parameters did not show consistent differences between pregnant and non-pregnant cows during the early post-insemination period (days 5–21). In contrast, changes in corpus luteum perfusion area and corpus luteum area from approximately day 19 post-insemination, together with increases in serum P4 concentrations from day 20 onward, were associated with differences between pregnant and non-pregnant animals. These findings suggest that vascular and morphological ultrasonographic parameters of the corpus luteum may provide earlier functional indicators of luteal status during early pregnancy compared with uterine artery spectral Doppler measurements or progesterone evaluation alone. As a non-invasive, repeatable, and cost-effective approach, B-mode and Doppler ultrasonography may offer practical advantages for monitoring early reproductive dynamics, particularly in herd management. The combined evaluation of corpus luteum perfusion area and corpus luteum area may potentially contribute to earlier identification of non-pregnant animals. Future research should focus on validating these findings in larger populations and under different management conditions, as well as integrating multimodal ultrasonographic and endocrine markers to refine early pregnancy assessment strategies.

## Figures and Tables

**Table 1:** Pearson correlation coefficients (r) among corpus luteum (CL), uterine, uterine artery parameters and progesterone (P4) in pregnant and non-pregnant cows.

Parameter Pair	Pregnant	Non-Pregnant
CL-MGV/UT-MGV	0.576 **	0.385 **
CL-MGV/CL-A	0.345 **	0.620 **
CL-A/CL-PA	0.343 **	0.700 **
CL-MGV/CL-PA	0.441 **	0.578 **
CL-A/P4	0.288 **	0.540 **
CL-PA/P4	0.137	0.248 *
P4/UT-MGV	−0.127	−0.366 **
P4/Uterine artery area	−0.174	0.006
P4/Max velocity	0.151	0.362 **
P4/Mean velocity	0.179	−0.057
P4/Flow volume	−0.007	−0.003
P4/Resistance index	0.006	0.279 **
P4/Pulse number	−0.054	−0.262 **

MGV: mean gray value, CL-MGV: corpus luteum mean gray value, CL-A: corpus luteum area, CL-PA: corpus luteum perfusion area, UT-MGV: uterine mean gray value, P4: progesterone concentration. * *p* < 0.05; ** *p* < 0.01.

## Data Availability

The original contributions presented in this study are included in the article. Further inquiries can be directed to the corresponding author.

## Abbreviations

The following abbreviations are used in this manuscript:

GnRH	Gonadotropin-releasing hormone
ROIs	Regions of interest
CL-MGV	Corpus luteum mean gray value
UT-MGV	Uterine mean gray value
CL-HOM	Corpus luteum homogeneity
UT-HOM	Uterine homogeneity
CL-PA	Corpus luteum perfusion area
CL-A	Corpus luteum area
P4	Progesterone
